# A Rare Intrathecal Pump Complication Caused by Prolonged Seroma Leading to a Potential Pump Pocket Fill: A Near Miss

**DOI:** 10.7759/cureus.48651

**Published:** 2023-11-11

**Authors:** Teddy Gerges, Angelina Mavropoulos, Danielle Levin, Thuan Dao, Martin Acquadro

**Affiliations:** 1 Anesthesiology, St. Elizabeth's Medical Center, Boston, USA; 2 Anesthesiology, New York University, New York, USA

**Keywords:** point-of-care drug screen test, pump interrogation, pump pocket fill, prolonged intrathecal pump pocket seroma, intrathecal pump complications

## Abstract

Intrathecal drug delivery systems have been used with increasing frequency in patients with chronic intractable pain. Common complications of intrathecal drug delivery systems include surgical bleeding, spinal cord injury, fractured or migrated catheter, meningitis, pump failure, granuloma formation, cerebral spinal fluid leak, and hygroma formation. We present a rare near-miss case that could have led to the inadvertent filling of an intrathecal pump pocket with a high concentration of narcotic and local anesthetic. This situation arose due to the discovery of a prolonged intrathecal pump pocket seroma during a routine maintenance and refill procedure.

## Introduction

In the United States, pain causes a staggering economic toll, estimated at $100 billion annually, while also inflicting significant emotional and psychological burdens on patients and their caregivers [1]. Since the identification of opioid receptors in the substantia gelatinosa in 1973 [2], intrathecal drug delivery systems have seen growing use, catering to patients dealing with both cancer-related and non-malignant pain. Despite the substantial initial cost associated with these systems, a 2002 Canadian study suggested that long-term intrathecal drug delivery proves cost-effective. Beyond the economic considerations, these systems offer the potential to mitigate systemic side effects and enhance the quality of life for patients. However, as with many invasive procedures, intrathecal drug delivery carries inherent risks related to surgical techniques, intrathecal catheter or mechanical failures, and pharmacological errors [2,3]. Prolonged seroma formation within the pump pocket, although rare, presents a unique complication that can inadvertently trigger erroneous feedback, potentially leading to an undesired pump pocket fill.

## Case presentation

**Figure 1:**
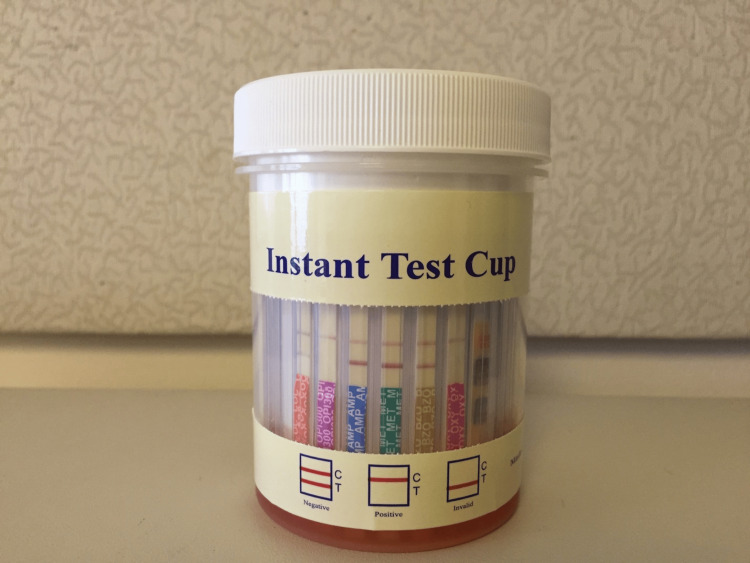
The aspirated fluid was tested using simple point-of-care urine tests and was found to be positive for morphine and oxycodone.

A 65-year-old male presented to the pain center for his routine intrathecal pump interrogation, maintenance, and refill. His medical history included hepatitis C, knee osteoarthritis, failed back syndrome, and anxiety. His usual intrathecal pump regimen comprised morphine 40 mg/mL and bupivacaine 30 mg/mL at a basal rate of 23.22 mg/day of morphine and 18.57 mg/day of bupivacaine, supplemented with oxycodone 5 mg every 8 hours for breakthrough pain. After seven stable years on this regimen, the patient's pump was replaced due to its end-of-life status. Post-replacement, he experienced colitis and stress-induced cardiomyopathy, necessitating a brief hospital stay. Two months later, he returned for a routine pump interrogation and refill without complications. However, five months post-pump replacement, during a refill, a significant discrepancy emerged between the interrogated and aspirated medication levels, raising concerns. During the pump interrogation, we verified a residual medication volume of 2.4 mL. The patient was positioned supine, and the pump site exhibited no signs of redness or swelling. After thorough sterile preparation and draping, we identified the access port using a prepackaged template. A 22 G Huber needle, connected to a clamped extension tube, was inserted beyond the skin, resulting in the aspiration of 10 mL of serous fluid. This volume inconsistency with the previously interrogated amount raised concerns. To confirm the fluid's origin and consistency, we decided to conduct a rapid on-the-spot test, readily available in our pain clinic, using a point-of-care urine drug screen cup, which visually detects the presence or absence of opioids. Surprisingly, once we poured the aspirated fluid into the cup, it tested positive for both oxycodone and morphine, indicating that it could not have originated from the pump chamber, which should contain only morphine (Figure 1). Consequently, we aborted the procedure, removed the needle, and transferred the patient to the fluoroscopy suite for a pump refill under X-ray guidance. With fluoroscopic assistance, around 2 mL of residual medication was aspirated, and 40 mL of medication consisting of a drug mix of morphine 40 mg/mL and bupivacaine 30 mg/mL was successfully refilled without any complications.

## Discussion

Intrathecal drug delivery is increasingly embraced as an option for patients who have exhausted conservative pain therapies. Common complications are associated with surgical technique, mechanical issues, and medication properties [1,3]. These encompass surgical bleeding, spinal cord injuries [4], catheter problems such as a fractured or migrated catheter [5-7], meningitis [8], pump failures, granulomas [9], cerebrospinal fluid leaks, hygromas, and pump pocket seromas. Rare but severe complications include cases such as inferior epigastric erosion and transverse myelitis associated with specific bacteria [10]. While seromas around the pump pocket are typically self-limiting lasting up to 2 months [1], prolonged seromas can lead to infections necessitating a fluid analysis, intravenous antibiotics, and pump removal. These fluid accumulations can induce discomfort and impede the necessary fibrosis process for securing the pump. Fortunately, such seromas usually resolve on their own, with the aid of a simple abdominal binder promoting comfort and healing. In the case at hand, the absence of fever or redness at the pump site ruled out the suspicion of an infected seroma. A substantial disparity between the interrogated and aspirated fluid volumes prompted us to question the source of the aspirated fluid. The considerably higher volume raised doubts about it originating from the pump chamber, as such a discrepancy would imply a significant pump malfunction. We conducted a straightforward point-of-care drug screen test, readily available in our clinic, on the fluid. The test revealed the presence of both morphine and oxycodone (Figure 1), contrary to the expectation of finding only morphine, given the patient's regimen of receiving morphine via the intrathecal pump and oxycodone orally. This outcome strongly suggested that the aspirated fluid did not originate from inside the intrathecal pump reservoir.

The extended presence of a seroma in this patient can be attributed to a thorough assessment of his clinical history, particularly his chronic hepatitis C and the occurrence of stress-induced cardiomyopathy. Patients with chronic hepatitis C can experience hyperdynamic cardiomyopathy and a reduction in synthetic liver function. Anesthesia has a moderate impact on hepatic blood flow, resulting in mild elevations in liver enzymes and a reduction in serum proteins essential for immune system function, nutrient metabolism, and drug processing [11]. In individuals with compromised liver function, whether due to chronic hepatitis C or other factors, the effects of anesthesia, whether general, spinal, or epidural, are more pronounced. Seromas, similar to the formation of ascites, can develop and persist beyond the expected timeframe due to decreased oncotic pressure resulting from reduced albumin levels.

Stress-induced cardiomyopathy, also known as takotsubo cardiomyopathy, is characterized by the apical ballooning of the left ventricle and a temporary decline in cardiac function triggered by extreme physical or emotional distress. Excessive catecholamines can promote sodium and water reabsorption in the kidneys, leading to extracellular fluid expansion and the persistence of seromas in potential spaces. Additionally, the abnormal Starling force caused by decreased forward flow, increased venous capillary pressure, and reduced plasma oncotic pressure may exacerbate fluid extravasation and seroma formation. Given the patient's post-operative experience with stress-induced cardiomyopathy, it is theoretically possible that he had not fully recovered from this condition, contributing to the prolonged seroma.

## Conclusions

This case report underscores the occurrence of a rare yet significant complication associated with implantable intrathecal drug delivery systems: the persistence of a seroma within the pump pocket for more than five months following surgery. It highlights the importance of clinical vigilance and the necessity to align a patient's clinical condition with their medical history and physical examination findings. In cases where discrepancies in clinical data arise, healthcare providers must remain attentive and draw connections between a patient's current state and their past medical history. This practice can lead to the early identification of unusual conditions or complications, potentially averting serious consequences. Furthermore, this case serves as a reminder of the potential dangers of the inadvertent delivery of a large volume of medication into the pump pocket. Such an event could result in severe respiratory depression, as well as neurological and cardiac toxicity due to local anesthetic systemic toxicity. Therefore, it underscores the need for meticulous attention to detail and adherence to established protocols during medical procedures. Lastly, this report emphasizes the importance of considering and investigating prolonged seroma formation when suspected. Although uncommon, the development of persistent seromas is possible in any patient, regardless of specific medical history or predisposing factors. Healthcare providers should utilize readily available tests and imaging modalities within clinical settings to promptly diagnose and address this issue. Doing so can prevent potentially catastrophic outcomes and ensure the well-being of patients undergoing intrathecal drug delivery procedures.
